# Clearing the Backlog: Trichiasis Surgeon Retention and Productivity in Northern Ethiopia

**DOI:** 10.1371/journal.pntd.0001014

**Published:** 2011-04-05

**Authors:** Esmael Habtamu, Saul N. Rajak, Teshome Gebre, Mulat Zerihun, Asrat Genet, Paul M. Emerson, Matthew J. Burton

**Affiliations:** 1 London School of Hygiene and Tropical Medicine, London, United Kingdom; 2 The Carter Center, Addis Ababa, Ethiopia; 3 The Amhara Regional Health Bureau, Bahir Dar, Ethiopia; 4 The Carter Center, Atlanta, Georgia, United States of America; University of California San Francisco, United States of America

## Abstract

**Background:**

In 2006 there were an estimated 645,000 people in Amhara, Ethiopia, with trachomatous trichiasis (TT) who needed surgery. Despite an extensive integrated eye care worker training programme (IECW) and robust support for TT surgical services, productivity has not reached targets. We investigated why surgeon productivity was below target.

**Methodology/Principal Findings:**

Confidential interviews were conducted in person with TT surgeons trained from 24 selected districts in Amhara Region and their supervisors. Determinants of attrition and productivity were investigated. We interviewed 225 people who had received IECW training; 139 (59%) had subsequently changed career/job. Staff retention was associated with good road access to their health centre, mobile telephone network and a shorter time from initial training. Amongst the 94 IECW still working in the programme, the average number of patients operated was 41/year, which was mostly (86%) done through outreach campaigns and only 14% of cases were performed in the static facilities where they routinely worked. Spot checks were made of surgical instruments and consumables: only 3/94 IECW had the minimum instruments and consumables to perform surgery. The main barriers to operating were lack of time, shortage of consumables, lack of patients, lack of support and equipment problems. Very few IECW received ongoing supervision or active management.

**Conclusions/Significance:**

Surgeon attrition rates are high. Vertical surgery campaigns were effective in treating large numbers of cases, whilst static-site service productivity was low. Good health system management is key to building a well-staffed and well-run service.

## Author Summary

Blindness from trachoma is caused by the abrasive effect of trichiasis (in-turned eyelashes). Surgery is performed to correct this anatomical abnormality, and prevent blindness. Despite the progress made in many regions in controlling the active/infectious stages of this disease, the global prevalence of trichiasis remains about the same. Current surgical activity is barely keeping abreast of incident trichiasis; this is undermining all the other efforts to control trachoma. We examine staff retention and productivity in Amhara Region, Ethiopia, where many hundreds of health care workers have been trained to perform the surgery. We found that the majority of people trained to do the surgery are now not in a position to do so. Amongst the sub-set that is still active within the programme, surgical productivity is low, with most surgery performed during “outreach campaigns” Insufficient surgical instruments and consumables were frequent problems. Strengthened health-systems management can overcome many of the problems that are holding back the delivery of an effective trichiasis surgical service.

## Introduction

Trachoma is the leading infectious cause of blindness worldwide [1]. Repeated ocular infection with *Chlamydia trachomatis* causes chronic conjunctival inflammation (active trachoma), which leads to conjunctival scarring. The scarring distorts the upper eyelid causing entropion and trichiasis (TT). This results in corneal abrasion, scarring and ultimately, blindness if left untreated. Trichiasis can also cause pain from the eyelashes rubbing on the cornea. About 1.3 million people are blind and a further 6 million people have severe visual impairment from trachoma [1]. It is estimated that 8.2 million people have un-operated TT.[2] Ethiopia has the largest burden of trachoma in Africa (30% of the total) with approximately 10 million cases of active disease and 1.2 million cases of TT (many of which are bilateral) [2], [3]. Within Ethiopia, Amhara region has a disproportionately large burden of trichiasis, with an estimated backlog of 645,000 un-operated cases (2006) [4].

The World Health Organisation (WHO) is leading an alliance for the Global Elimination of Blinding Trachoma by the year 2020 (GET2020) through the implementation of the SAFE Strategy [5]. This involves the provision of **S**urgery to correct trichiasis, **A**ntibiotic distribution to treat chlamydial infection, **F**acial cleanliness and **E**nvironmental improvements to reduce transmission and re-emergence of the infection within treated communities.

The SAFE Strategy is being implemented throughout Ethiopia by governmental and non-governmental organizations. In Amhara the trachoma control programme is a collaboration between the Amhara National Regional Health Bureau (ANRHB) and the Lions-Carter Center SightFirst Initiative. The programme started in 24 districts between 2001 and 2004. In common with many other trachoma control programmes, the Amhara trachoma control programme has trained non-ophthalmologists to perform TT surgery. Generally, these individuals, referred to as Integrated Eye Care Workers (IECW), are drawn from various health facilities and provided with up to 4 weeks training in TT surgery. At the end of training each surgeon has to pass the WHO “Final Assessment of Trichiasis Surgeons” certification process before being allowed to practice [6]. The results of surgery by this cadre have previously been found to be comparable with that of ophthalmologists [7]. After completing the training the TT surgeons return to their respective health facilities, where they are expected to perform TT surgery amongst their other duties. It is planned for each district to have between two and four IECWs, supported by an ANRHB district blindness prevention officer and a zonal project coordinator. Surgery is currently provided either through the “static-site” health facilities or through periodic campaign programmes.

From the prevention of blindness perspective, the large backlog of un-operated TT is a cause for major concern. Despite the efforts of many, the most recent global estimates do not show a decline in the number of people needing TT surgery, suggesting that current surgical activity is only just keeping pace with incident trichiasis and not reducing the overall backlog [2], [8]. In Amhara, since the 2006 survey, around 100,000 TT surgeries have been performed, which represents about 16% of the estimated backlog; although it is unknown how many incident cases of TT may have developed during these years. The programme has trained, equipped, and provided financial and logistical support to 714 IECWs from throughout the region since 2001, 129 of whom were trained and certified in 2009 and 2010. This number of trained TT surgeons was anticipated to be able to successfully provide counselling and corrective surgery to all consenting patients to eliminate the surgical backlog by 2015. Despite the program being the most productive in the world in terms of patients operated, accounting for around a third of all TT surgeries reported at the WHO Global Alliance meetings in 2007, 2008 and 2009, output is lower than expected and lower than that required to eliminate the backlog. We conducted a study to firstly evaluate the amount and reasons for attrition of IECWs from the programme and secondly to assess the current productivity of the surgical service to identify ways to better select trainees and ultimately to improve service delivery.

## Methods

### Ethics Statement

This study was approved by the National Health Research Ethics Review Committee of the Ethiopian Ministry of Science and Technology, the London School of Hygiene and Tropical Medicine Ethics Committee and the Emory University Institutional Review Board. Written informed consent was required for participation in this study. It was conducted in accordance with the principles of the Declaration of Helsinki.

### Study Participants

This study included the 24 districts of West Amhara where The Carter Center assisted trachoma control programme has been operational for five or more years. We compiled a list of all individuals from these districts who received IECW training to perform TT surgery between 2001 and 2009. IECWs were contacted by telephone to arrange a face-to-face interview, wherever possible. If an IECW could not be contacted directly, their district manager and other colleagues at their last known work location were approached to help trace them.

### Structured Interviews

Confidential structured interviews were conducted with all study participants. Questionnaires were pre-tested on 10 surgeons and 2 managers in non-study districts. The questionnaires were administered in Amharic by one trained interviewer (EH), who travelled to the participant's place of work. Individuals working in very inaccessible areas were interviewed by telephone. Trichiasis surgeons were sub-divided into two groups: (1) “surgeons who were still in the programme”, defined as being in posts where they could potentially carry out TT surgery, and (2) “surgeons who were lost from the programme”, defined as no longer in positions where they could perform TT surgery. Separate questionnaires were developed for these two groups. A third questionnaire was used to interview the district blindness prevention officer. For surgeons who were still in the programme we asked specific closed questions on: demography, road access to the health centre (where good was defines as accessible for 9+ months a year), mobile phone network coverage, facilities, training, supervision, current surgical practice, surgical output, and the availability of surgical equipment and consumables. Good access to consumables was defined as all consumables being available for at least 2/3 of the time. We also asked questions to elicit their opinions about barriers to performing surgery and their suggestions on how to improve the productivity of the service. For each barrier that the individual mentioned we asked them to grade its severity weighting using a five point scale: 0 “Never”, 1 “Rarely”, 2 “Sometimes”, 3 “Often” and 4 “Always”. During the visit to the health facility the availability, number and condition of instruments and consumables for TT surgery were assessed directly. For surgeons who were lost from the programme, we also collected data on their current job and reasons for no longer operating. The district blindness prevention officer questionnaire asked about the current number of TT surgeons in the district, their supervision, current surgical output and barriers to productivity. The data collection was carried out between February and May 2010.

### Data Analysis

Data were double entered and verified in MS Access and analyzed in STATA 11. Descriptive data and univariate odds ratios were calculated to assess potential risk factors for attrition from the programme and current productivity within the programme. Multivariable logistic regression models were developed to test the independent significance of various factors. To determine the relative importance of each barrier identified by the surgeons we summed the severity weighting score given by each surgeon who mentioned the specific barrier.

## Results

### Amount and reasons for attrition

**Figure 1:**
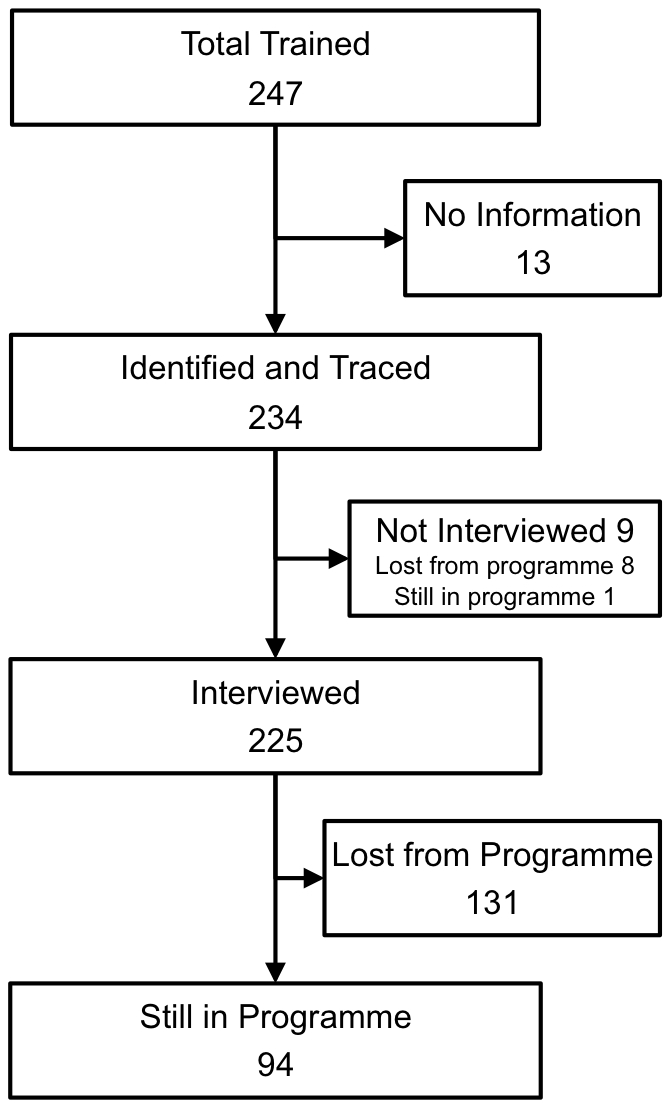
Trichiasis surgeons trained in West Amhara Region, Ethiopia. Flow diagram showing the numbers trained, identified, interviewed and still working in the TT surgery programme.

Between 2001 and 2008, 247 TT surgeons were recorded as having been trained from the 24 districts of West Amhara. We attempted to trace and interview all of these individuals. Figure 1 illustrates the numbers that were traced, interviewed and still in posts where they could perform TT surgery (still in programme). Of the 234 people identified and traced, 139 (59.4%) were no longer in a position to perform TT surgery (attrition). We interviewed 131/139 of these people and asked about their current position: 72 (54.9%) had moved to another more senior post within the public health system, 39 (29.8%) were receiving long-term training (>1 year), 10 (7.6%) were working for an NGO and 10 (7.6%) were working in the private sector. Of those who had been lost from the programme 70/131 (53%) said that another person based at their original health facility took over their TT surgery role.

**Figure 2:**
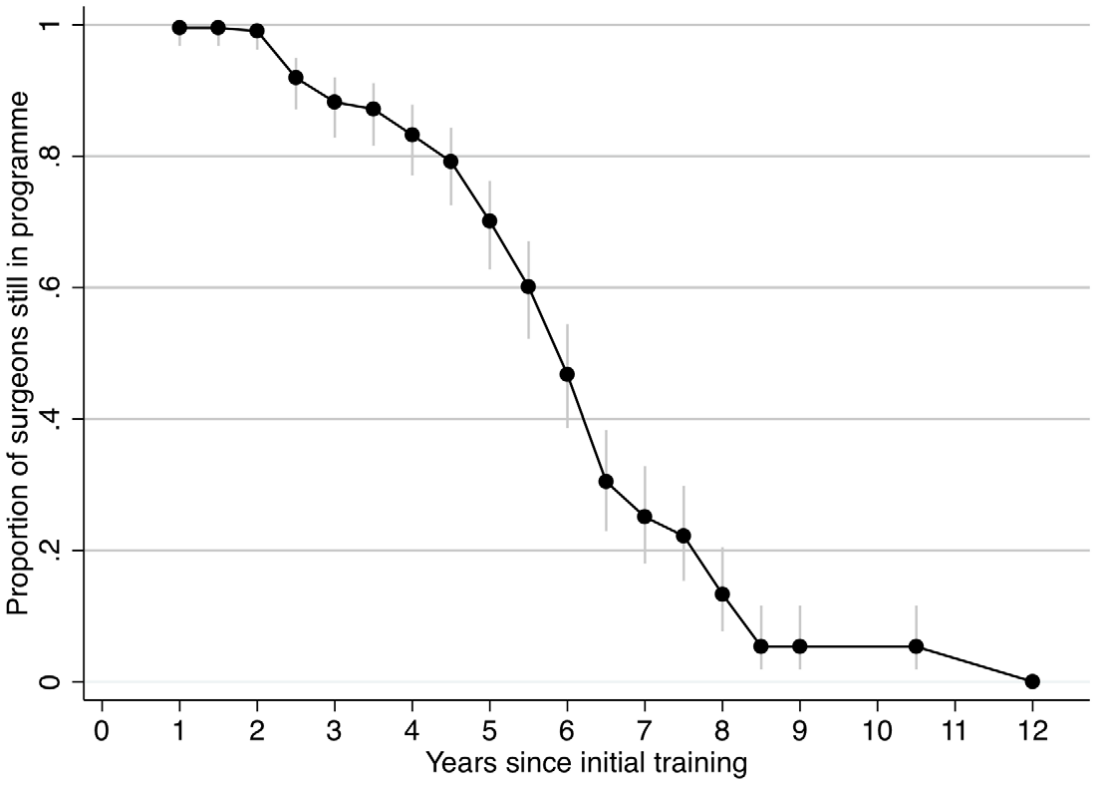
Survival curve for surgeons still working in the Amhara TT surgery programme. This is with respect to the time since they received their initial training. Vertical bars represent the 95% confidence intervals.

The major characteristics of the 225 interviewed surgeons are presented in Table 1, subdivided into those who are still in the programme (“IN”) and those not (“LOST”). There were significant univariate associations between continuing to be in the programme and: female sex, based in a health facility with good road access, mobile phone coverage and electricity supply. Individuals who had completed more than the required minimum of 30 TT operations during their training were less likely to remain in the programme. Individuals who were currently working in the programme had been trained more recently than those lost (Figure 2). There was no statistically significant difference in: age, marital status, being a parent or reason for training. The level of education or training received prior to TT surgery training did not make a significant difference to the attrition rate. In a multivariable logistic regression model for still being in the programme (Table 2) significant associations remained for: recent training, good road access and mobile phone coverage.

**Table 1:** Characteristics of the individuals trained in TT surgery in West Amhara Region, Ethiopia.

Variable	All (N = 225)		IN (N = 94)		LOST (N = 131)		OR	(95% CI)	P
	n	(%)	n	(%)	n	(%)			
**Age**, mean yrs (SD)	30.7	(6.5)	30.3	(6.9)	30.9	(6.1)			0.49*
**Sex** (female)	77	(34.2)	44	(46.8)	33	(25.2)	2.61	(1.48–4.60)	0.001
**Dependent children**	101	(44.9)	54	(57.5)	70	(53.4)	1.18	(0.69–2.01)	0.551
**Marital Status**									
Single	89	(39.6)	33	(35.1)	56	(42.8)	1	-	-
Married	130	(57.8)	58	(61.7)	72	(55.0)	1.37	(0.79–2.37)	0.267
Divorced	6	(2.7)	3	(3.2)	3	(2.3)	1.70	(0.32–8.90)	0.532
**Health centre facility**									
Good road	180	(80.0)	85	(90.4)	95	(72.5)	3.58	(1.63–7.86)	0.001
Water supply	159	(70.7)	72	(76.6)	87	(66.4)	1.66	(0.91–3.01)	0.099
Electric supply	126	(56.0)	69	(73.4)	57	(43.5)	3.58	(2.02–6.36)	<0.001
Mobile network	114	(50.7)	74	(78.7)	40	(30.5)	8.42	(4.54–15.6)	<0.001
**Number of lids operated during training**									
0–10 lids	33	(14.7)	19	(20.2)	14	(10.7)	1	-	-
11–30 lids	108	(48.0)	44	(46.8)	64	(48.8)	0.51	(0.23–1.11)	0.92
31+ lids	84	(37.3)	31	(33.0)	53	(40.5)	0.43	(0.89–0.98)	0.044
**Training selection criteria**									
Self requested	4	(1.8)	2	(2.1)	2	(1.5)			
Circumstantial	142	(63.1)	61	(64.9)	81	(61.8)			
Ability	40	(17.8)	16	(17.0)	24	(18.3)			
Unknown	39	(17.3)	15	(16.0)	24	(18.3)			
**Highest training/education level, prior to TT training**									
Junior nurse	12	(5.3)	5	(5.3)	7	(5.3)			
Diploma nurse	184	(81.8)	75	(79.8)	109	(83.2)			
BSc nurse	29	(12.9)	14	(14.9)	15	(11.5)			
**Refresher training**	107	(47.6)	52	(55.3)	55	(42.0)	1.71	(1.00–2.9)	0.049
**Time since training**									
Mean, months (SD)	55	(25)	46	(27)	61.5	(22)			<0.001*

Subdivided into those who are (IN) and are not (LOST) working in the programme. Univariate associations are shown for continuing to work in the programme.

*Unpaired t-test.

**Table 2:** Multivariable logistic regression model for remaining within the TT surgery programme, West Amhara Region, Ethiopia.

Variable	OR	(95% CI)	P value
Sex (female)	1.44	(0.69–3.01)	0.335
Mobile telephone network	10.04	(4.71–21.4)	<0.001
Good road access	3.96	(1.31–12.0)	0.015
Time from training			
<2 ears	1.00	-	-
2–4 years	0.05	(0.01–0.22)	<0.001
4–6 years	0.03	(0.01–0.14)	<0.001
>6 years	0.02	(0.01–0.11)	<0.001

### Static-site surgical services

Of the 94 TT surgeons still in the programme, 51 (54%) had performed static-site surgery during 2009/10 on a total of 531 cases. The mean number of static-site cases performed by the 51 active surgeons was 10.4 cases/surgeon/year (95% CI 5.7–15.1). Characteristics of all 94 surgeons are shown in Table 3, sub-divided by whether or not they had performed any static-site surgery during 2009/10. Performing static-site surgery was associated (univariate) with: good access to consumables, having an annual surgical plan and advertising the service locally. There was no difference in age or sex. Fifty-one (54.3%) surgeons had received some refresher training since their original TT surgery training. However, this was not associated with increased static-site surgical activity. The refresher training only involved a practical component for 19%. Supervision was infrequent (10/78, 12.8%) and not associated with increased static-site surgery productivity (Chi2, p = 0.399). In a multivariable logistic regression model performing static-site surgery was independently associated with both good access to consumables and advertising the service (Table 4).

**Table 3:** Surgical productivity in static-sites.

Variables	All (N = 94)		Operating (N = 51)		Not Operating (N = 43)		OR	(95% CI)	P
	n	(%)	n	(%)	n	(%)			
**Age**, mean (SD)	30.3	(6.9)	31.1	(7.1)	29.3	(6.5)			0.214*
**Sex**, female	44	(46.8)	23	(45.1)	21	(48.8)	0.86	(0.38–1.94)	0.717
**Surgical plan**	48	(51.1)	31	(60.8)	17	(39.5)	2.37	(1.03–5.44)	0.042
**Consumables**	30	(31.9)	25	(49.0)	5	(11.6)	7.31	(2.48–21.6)	<0.001
**Advertise**	49	(52.1)	36	(70.6)	13	(30.2)	5.54	(2.28–13.4)	<0.001
**Number of lids operated during training**									
0–10	19	(20.2)	8	(15.7)	11	(25.6)	1	-	-
11–30	44	(46.8)	22	(43.1)	22	(51.2)	1.38	(0.46–4.01)	0.565
31+	31	(33.0)	21	(41.2)	10	(23.3)	2.89	(0.89–9.41)	0.079

Factors associated with performing static-site TT surgery for 94 TT surgeons in the programme (univariate OR).

*Unpaired t-test.

**Table 4:** Multiple logistic regression model for performing any static-site surgery.

Variable	OR	(95% CI)	P value
Good access to consumables	8.42	(2.57–27.5)	<0.001
Advertise programme	6.31	(2.34–17.1)	<0.001

Spot-checks of instruments for TT surgery revealed that only 25/94 (26.6%) had a complete standard 7-piece TT set, and a further 21/94 (22.3%) had the minimum 5-piece TT set. Being supervised was not associated with improved availability of a minimum 5-piece TT set (Chi2, p = 0.199) Spot-checks of consumables available to the TT surgeons in their health centre found that only 14/94 (14.9%) had the minimum necessary to perform surgery. Only 3/94 (3.2%) had both the minimum 5-piece TT set and the minimum consumables available to perform surgery on the spot-check day. There was no difference in the age, gender or time from training between those surgeons (94 active only) who did or did not have sufficient equipment or consumables.

### Surgical campaign services

During 2009/10 52/94 (55.3%) TT surgeons participated in occasional organised surgical campaigns. Altogether 3319 cases were operated in campaigns by 52 surgeons; mean 64 cases/surgeon/year (95%CI 41.0–86.6). No surgery was done at all during 2009/10 by 22/94 (23%) TT surgeons. Overall, the mean number of surgeries done by all 94 surgeons still in the programme was 41 cases/surgeon/year during 2009/10 (outreach and static-site combined). There were generally not problems with consumable supplies for surgical campaign services as these were stocked directly from the central supply store.

### Barriers to doing surgery

Surgeons reported ten separate issues that can be barriers to performing surgery, particularly in the static-site environment. These are ranked in order of frequency in Table 5. Severity weights were calculated based on the subjective five-point severity score, for each barrier listed. The most important were: poor access to consumables, lack of time, patients not presenting, limited senior support and equipment problems. Senior support included that received from health centre managers, district blindness prevention officers and the supporting NGO.

**Table 5:** Barriers to performing surgery.

Barrier	ALL (N = 225)			IN (N = 94)			LOST (N = 131)		
	n	(%)	Wt	n	(%)	Wt	n	(%)	Wt
Consumables: none, incomplete	102	(45.3)	292	54	(57.4)	168	48	(35.6)	124
Lack of time/Other responsibilities	101	(44.9)	285	38	(40.4)	108	63	(48.1)	177
Lack of patients attending	95	(42.2)	258	47	(50.0)	138	48	(36.6)	120
Lack of senior support	75	(33.3)	192	33	(35.1)	83	42	(32.1)	109
Surgical equipment: none, incomplete	64	(28.5)	204	42	(44.7)	134	22	(16.8)	70
Away from work place (sick, training)	61	(27.1)	136	22	(23.4)	48	39	(29.8)	88
No financial incentive	43	(19.1)	108	16	(17.0)	39	27	(20.6)	69
Problem with sterilising TT sets	26	(11.6)	65	6	(6.4)	16	20	(15.3)	49
No suitable place for operating	21	(9.3)	66	14	(14.9)	45	7	(5.34)	21
Inadequate training	2	(0.9)	8	2	(2.1)	8	0	(0)	0

Closed questions to all 225 interviewed surgeons, subdivided into those who are (IN) and are not (LOST) working in the programme. Number (n) and % of surgeons identifying a specific barrier. The weight (Wt) is the sum of the severity weighting score given by each surgeon who identified the specific barrier.

Surgeons who were still in the programme were also asked why they thought patients were not presenting. Reasons included: lack of patient awareness (43.8%), no static-site service (23.3%), reduced backlog (21.9%), patients want an expatriate surgeon (15.0%) and poor surgical quality (9.5%).

All 225 interviewed surgeons were asked to provide suggestions on how to improve the static-site surgical output, the top five suggestions were: improve patient awareness (64.0%), increase availability of consumables (33.8%), refresher training (30.2%), support and supervision (29.8%), and financial incentives (28.4%).

### Supervisor interviews

We interviewed 24 district blindness prevention officer (supervisors), one for each district. These are mid-level health workers, usually with a nursing background. These individuals had generally only been in post for a relatively short period (median 6.5 months, IQR 6-15 months). For seven supervisors trachoma/prevention of blindness activities was their main management responsibility. For the rest, trachoma was one of several disease control or management responsibilities, which also included malaria, TB, HIV and health extension workers. They reported supervising a median of 4 active surgeons in their district (range 1–10). All district supervisors reported losing TT surgeons, the number was proportional to their duration in post (p<0.001). The main reasons reported for the loss of surgeons were: transferred to different district (67), left government service (39), further education (35), and promotion (32). Surgical campaigns had been held in most districts within the last six months (16/24) and most supervisors felt that this was the better way to deliver the service (15/24). Most supervisors had an annual plan for TT surgery in their district (19/24). Supervision was variable: 10 had never met with any of their surgeons to discuss TT surgery, 11 had met with one or more surgeons in the last six months.

## Discussion

The backlog of un-operated TT is undermining the global effort to control blinding trachoma by the year 2020. In the Amhara National Regional State of Ethiopia, the most affected State in the most affected country, there are probably still more than 500,000 un-operated cases. The regional prevention of blindness plan has set a target of operating 100,000 cases/year. However, over the last three years the average number of patients operated per annum has been around 32,000. This is far below the target, despite more than 700 health care professionals having been trained to perform surgery. It is therefore important to understand the determinants of attrition and lower than planned productivity in order to address the unmet need.

Attrition from the surgery programme was common; more than half of those trained were no longer in positions where they performed TT surgery. The majority were lost because they had moved to other, generally more senior posts in the public health system. Men were more likely to be lost from the programme than women (possibly because they are more likely to be promoted) although this was not significant in a multivariable model. The working environment is important for staff retention: retention was associated with good road access, mobile telephone network and electricity (NS in model). These observations are consistent with studies from a variety of settings, which have found that working and living conditions as well as remuneration are key determinants in staff retention in low-income countries [9], [10].

Candidates for surgical training need to be carefully selected. Firstly, individuals should have an aptitude for practical procedures. This could be determined by assessment of basic skills such as suturing. Secondly, note should be taken of how long the individual is likely to remain in their current position. It is possible that more junior cadres, such as highly selected nursing auxiliaries or primary health care workers, might be less likely to relocate [9]. It is uncertain from available data whether refresher training improves retention, as the observed association between receiving refresher training and remaining in the programme is likely to be due to reverse causality. In other settings additional training interventions have been found to have only limited impact on health-worker performance [10].

Overall productivity was lower than anticipated: 41 cases a year against a target of 200 patients/surgeon/year amongst surgeons still in the programme. This figure compares favourably with that reported from Tanzania (22 cases/year), however, the size of the backlog in Amhara is much greater [11]. In common with prevention of blindness programmes in other countries, the Amhara programme is intended to be integrated within the general health system, enabling surgery to be provided along side routine activities on a day-to-day basis at static-site facilities. However, the vast majority of surgery performed in Amhara Region in recent years has been through vertically organised outreach campaigns. This raises important questions over the best delivery strategy for this service. It is likely that the quality of surgery is higher when surgeons are doing a greater volume. This tension between alternative approaches is seen in various situations where disease specific programmes try to integrate within the health system [12]. The most appropriate strategy will depend on a range of factors, but will probably need a combination of the two approaches. Unfortunately, there are no studies that can guide policy makers in the most effective delivery strategy.

We explored the reasons for low productivity through static-site services. Significant associations were found between performing surgery and reported good availability of consumables and advertising the service. Common reasons cited for not operating was lack of time due to other work activities and lack of patients. There are probably multiple reasons for patients with TT not self-presenting for surgery. The impression of the surgeons themselves was that this was at least in part related to a lack of patient awareness of the treatment option for TT, indicating the importance of improving health promotion messages. The surgeons also cited the lack of a functioning static site service as another leading reason for the low patient numbers. It is possible that allocating and protecting regular time for performing surgery, and advertising this to patients might help ensure that both surgeons and patients are prepared adequately. Refresher training usually only involved theory classes. It is possible that including a practical component might encourage people to do more surgery, particularly if they lack confidence in their surgical skills. Financial incentives may be a significant determinant of activity, although this was not particularly emphasised by the interviewed surgeons. A distinction between static-site and campaign surgery is that the latter attracts an additional payment. The provision of incentives is not without difficulties and unintended side-effects and has the potential to undermine the long-term sustainability of programmes [9]. Therefore, we feel that it is preferable that TT surgery is treated as a component of the specially trained health workers job description and is performed as a routine activity; rather than only when externally provided incentives are offered. It is important to note that the surgeon's view is only one of several perspectives in analysing the reasons for low productivity; the views of the patient and managers are also integral to the process of understanding the barriers and developing strategies to overcome these.

Since the beginning of this TT surgery programme more than 350 complete TT surgery kits have been supplied to staff working in the 24 districts. However, despite this significant financial investment only a minority of active surgeons had access to sufficient instruments to perform surgery. The programme buys large amounts of consumables each year to perform TT surgery. However, only a small number of surgeons had all the essential items necessary to perform TT surgery. When both instrument and consumable availability are considered together, only 3% of the active surgeons had what they needed to perform static-site surgery when a spot-check was performed. This situation is significantly worse than the subjective response given by surgeons on the availability of consumables. The Amhara programme receives significant external support and technical assistance. It is quite likely that the availability of instruments and supplies is worse in other programmes that have less support. The management of supply chains is a critical issue: no consumables – no surgery.

The role of supervision needs to be strengthened within this programme. Both the surgeons and the supervisors reported relatively limited contact with each other. After the initial training, few surgeons are ever observed operating again by a more experienced person. The campaign mode of delivery offers the advantage of occasionally bringing a few surgeons together and providing an opportunity to share expertise. Lessons from other health care situations in low and middle-income countries suggest that constructive supervision with audit can improve the quality of health care [10].

We did not evaluate the TT surgical activity of private clinics or the larger regional hospitals (which are not located within any of these 24 districts). However, our impression is that very little TT surgery is done through either. This study did not address the important issue of the quality and outcome of surgery. The results of TT surgery are known to vary significantly between surgeons, especially under operational conditions [13]. It is very unusual to find programmes or TT surgeon auditing their own results, partly because of the logistical challenge and expense of following up patients in remote settings months after the surgery is done.

It is likely that many of the issues documented in this study will be common to trachoma control programmes in other sub-Saharan African countries. Overall, the key determinants of productivity in this TT surgical service are its structure and management. The striking finding in this study was that the vast majority of surgery was done in the context of specially organised campaigns, which received additional financial and logistical support from an NGO. This runs counter to the prevailing trend of promoting the integration of health services. However, it seems unlikely that in the Ethiopian context that static site (integrated) services will be able to deal with the very large backlog of un-operated trichiasis. The appropriate selection and placement of surgeons influence whether the individual continues to work in the service. For ongoing activity within static-site facilities many of the major obstacles can be overcome with good active management, particularly in timetabling and supervision. Consumables are provided free of charge to the Amhara programme, however, many of the surgeons were not receiving what they needed, which could be improved with better management of supply chains. There is a need to address these and other health system management issues so that fewer people become needlessly blind from trachomatous trichiasis.
